# Multifractal Detrended Fluctuation Analysis of Human EEG: Preliminary Investigation and Comparison with the Wavelet Transform Modulus Maxima Technique

**DOI:** 10.1371/journal.pone.0068360

**Published:** 2013-07-03

**Authors:** Todd Zorick, Mark A. Mandelkern

**Affiliations:** 1 Department of Psychiatry, Greater Los Angeles Veterans Administration Healthcare System, Los Angeles, California, United States of America; 2 Department of Imaging, Greater Los Angeles Veterans Administration Healthcare System, Los Angeles, California, United States of America; 3 Department of Psychiatry and Biobehavioral Sciences, University of California Los Angeles, Los Angeles, California, United States of America; 4 Department of Physics, University of California Irvine, Irvine, California, United States of America; University of Zurich, Switzerland

## Abstract

Recently, many lines of investigation in neuroscience and statistical physics have converged to raise the hypothesis that the underlying pattern of neuronal activation which results in electroencephalography (EEG) signals is nonlinear, with self-affine dynamics, while scalp-recorded EEG signals themselves are nonstationary. Therefore, traditional methods of EEG analysis may miss many properties inherent in such signals. Similarly, fractal analysis of EEG signals has shown scaling behaviors that may not be consistent with pure monofractal processes. In this study, we hypothesized that scalp-recorded human EEG signals may be better modeled as an underlying multifractal process. We utilized the Physionet online database, a publicly available database of human EEG signals as a standardized reference database for this study. Herein, we report the use of multifractal detrended fluctuation analysis on human EEG signals derived from waking and different sleep stages, and show evidence that supports the use of multifractal methods. Next, we compare multifractal detrended fluctuation analysis to a previously published multifractal technique, wavelet transform modulus maxima, using EEG signals from waking and sleep, and demonstrate that multifractal detrended fluctuation analysis has lower indices of variability. Finally, we report a preliminary investigation into the use of multifractal detrended fluctuation analysis as a pattern classification technique on human EEG signals from waking and different sleep stages, and demonstrate its potential utility for automatic classification of different states of consciousness. Therefore, multifractal detrended fluctuation analysis may be a useful pattern classification technique to distinguish among different states of brain function.

## Introduction

While human electroencephalography (EEG) recordings have been utilized for clinical and research purposes since the 1920s, still much is unknown about the underlying neuronal dynamics responsible for scalp-recorded electric potential changes as a function of time [1], [2]. Based upon the physiological and conductive properties of the intervening scalp and skull, EEG electrodes are thought to record space-averaged electrical potentials representing synaptic activity of 10^8^–10^9^ cortical neurons, therefore with poor spatial resolution, but excellent temporal resolution compared to other neuroimaging modalities [2], [3]. Current clinical uses of EEG involve spectral analysis via Fourier transform, which can accurately decompose underlying signal frequencies of a stationary signal [1], .

However, many lines of investigation into the neuronal dynamics which underlie scalp-recorded EEG have opened up the possibility that other techniques, derived from statistical mechanics, may also be useful for the analysis of EEG signals [6]–[8]. EEG signals themselves have been reported to be highly non-stationary [9]. Direct recording of cortical neurons in animal cortices has provided convincing evidence for the presence of scale-free (self-affine) dynamics in the patterns of neuronal avalanches in cortical neurons [10]–[13]. Indeed, neuronal avalanches recorded in the cortex were also found to correlate with beta/gamma band EEG recordings in rodents [14]. Evidence of scale-free network activation has also been demonstrated utilizing functional magnetic resonance imaging, magnetoencephalography, and electrocorticography [7], [8], [15], [16]. This experimental evidence collected on neuronal dynamics is matched by theoretical observations demonstrating that information networks operating at a “critical” state (exhibiting scale-free or self-affine dynamics) tend to maximize information transmission [12], [17]. Therefore, traditional statistical methods of EEG analysis (e.g., spectral analysis via Fourier transform) may lose essential information about the neuronal dynamics underlying EEG signals, since these techniques would miss many properties inherent in nonstationary signals with self-affine dynamics.

Methods derived from statistical physics have been applied to the analysis of human EEG signals with a moderate degree of success [3], [7]. While the field is too broad to comprehensively review for the scope of this report, we will discuss one of the most frequently utilized methods for analyzing time series with scale-free dynamics, the detrended fluctuation analysis (DFA) [18], which has also been extensively utilized on human EEG signals [16], [19]–[23]. DFA is an efficient technique to assess monofractal power-law scaling in the presence of nonstationary trends in the data [24]. DFA (combined with frequency filtering) has been shown to be useful as a tool to characterize differences in brain states in depression [21], sleep stages [19], and in hypnosis [20]. However, the application of DFA to EEG has also been generally limited to frequency-filtered portions of the EEG signal, due to the presence of different scaling regimes in the unfiltered signals [8].

While many natural self-affine systems exhibit power-law behaviors well described with a single fractal exponent, a more complicated version of self-affine systems was first described by Mandelbrot [25], [26], where the fractal nature of the system is better described as an interwoven series of different fractal exponents, or a “multifractal” [27]–[29]. Several different interpretations of the physical meaning of multifractal analysis have been proposed, but we give here a description provided by Mandelbrot and colleagues [30], [31]. Briefly, the multifractal spectrum is a plot of the fractal dimension of a set of instants (“D(h)”, with values ≤1) versus the corresponding values of Hölder exponents (“h”) for those instants [30], [31]. The local Hölder exponent (which we shall denote *h(t)*) measures the local regularity of a given time series process *X(t)* with stationary increments [30], [31]:
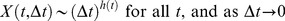
(1)


Here *X(t,* Δ*t) = X(t+*Δ*t)- X(t).* For a true multifractal process, h will exhibit a wide range of values, whereas for monofractal process, h will approach a single value, such that the degree of multifractality of a given series can be estimated via the range of the h values (cf. [32]). The resultant multifractal spectrum, with Hölder exponents plotted as the abscissa, and the fractal dimensions as the ordinate, typically approximates a truncated inverted parabola [32], [33].

The most widely utilized method for analyzing multifractal time series has perhaps been the wavelet transform modulus maxima (WTMM) technique [34], which has been successfully applied to many natural systems [32]. WTMM has been utilized in human EEG to assess differences among stages of sleep [35], [36], and among different psychiatric conditions [37], therefore showing evidence of its utility in human EEG research.

DFA has been extended to include a multifractal formalism, called multifractal DFA (MF-DFA), which combines the ease of computation inherent in the DFA technique with the ability to assess for multifractality in time series [24], [33]. There have been successful applications of MF-DFA to geophysics and to the biology of ion current fluctuations [38]. In several head-to-head comparisons of MF-DFA with WTMM on the same datasets, both methods have been found to be reliable, though MF-DFA has tended to produce more consistent results [33], [39].

To our knowledge, however, MF-DFA has not yet been applied to EEG signals. The objective of this study is to investigate the use of MF-DFA as a tool to assess multifractality in human EEG, using sleep-stage data from a publicly available database. We report here the use of MF-DFA on human EEG signals, and show that human EEG is well-modeled by a multifractal process when compared with numerical simulations of both monofractal and multifractal processes. Next, we compare WTMM and MF-DFA on the same EEG data, and show that MF-DFA tends to have lower variability on several multifractal spectral indices. Finally, we perform several tests of MF-DFA as a tool to characterize different sleep stages among subjects, and show that even short EEG tracings of 30 s–1 m can have robust differences in multifractal spectra. Taken together, these data provide support for the possibility that analysis of EEG by MF-DFA may be a valuable tool in the automatic characterization of changes in brain and/or consciousness states.

## Methods

### Ethics

Approval for this study utilizing data from a publicly available, deidentified database was provided by the local VA West Los Angeles Institutional Review Board.

### Database

Single channel EEG recordings with sleep stage annotations were downloaded from the MIT-BIH polysomnographic database (slpdb) from www.physionet.org (sampled at 256 Hz) [40], [41]. The list of subject numbers and data utilized is provided in Table 1. Tracings were selected randomly based only upon relative lack of obvious movement artifacts. Both contiguous and non-contiguous tracings were joined together in 1 m (n = 15000) segments that were annotated to be in the same consciousness state. Of the 16 possible subject records, only 14 had usable waking EEG tracings of >1 m in length (Table 1).

**Table 1:** List of subject numbers and data utilized.

Subject	8 min waking/sleep 2 data?	recording site		1 min sleep stage data:			
					waking	sleep 1	sleep 2		sleep 3		REM	
S1	yes		C4-A1		yes	yes	yes		no		yes	
S2	yes		O2-A1		yes	no	yes		no		yes	
S3	yes		C3-O1		yes	yes	yes		no		yes	
S4	yes		C3-O1		yes	yes	yes		yes		yes	
S14	yes		C3-O1		yes	yes	yes		yes		yes	
S16	yes		C3-O1		yes	no	yes		yes		yes	
S32	yes		C4-A1		yes	yes	yes		yes		no	
S37	yes		C4-A1		yes	no	yes		no		no	
S41	yes		C4-A1		yes	yes	yes		yes		yes	
S45	no		C3-O1		no	no	yes		yes		yes	
S48	yes		C3-O1		yes	yes	yes		no		yes	
S59	yes		C3-O1		yes	no	yes		yes		yes	
S60	yes		C3-O1		yes	yes	yes		no		yes	
S61	yes		C3-O1		yes	yes	yes		yes		yes	
S66	yes		C3-O1		yes	no	yes		no		no	

### Multifractal Time Series Analysis

Code for MF-DFA was written in the R programming language [R Core 42] following the original description of the technique [33]. While various ranges of q were tested, multifractal spectra were most consistent with the range of −5≤q≤5 (data not shown). Similarly, while higher-order polynomial detrending produced equivalent results, overall the spectra were well-characterized with a linear detrending procedure, which was thus exclusively utilized for this study (MF-DFA1; data not shown).

Code written for WTMM was downloaded and used as described: http://www.physionet.org/physiotools/multifractal/ [40]; the code was written to follow the original description of the technique [34]. To complement the MF-DFA analysis (see above), −5≤q≤5 was also used to generate multifractal spectra, with intervals of 0.2 units of q, such that multifractal spectra from both techniques were of the same length.

In the following, we use the *h* vs. *D(h)* naming convention, where *h* is the Hölder exponent (abscissa) of a fractal subset and *D(h)* (ordinate) is the corresponding fractal dimension, after [43], rather than the *α* vs *F(α)* convention as in [24]. We refer to the graphs of *D(h)* vs *h* as MF-DFA and WTMM spectra, depending upon the method used.

**Figure 1:**
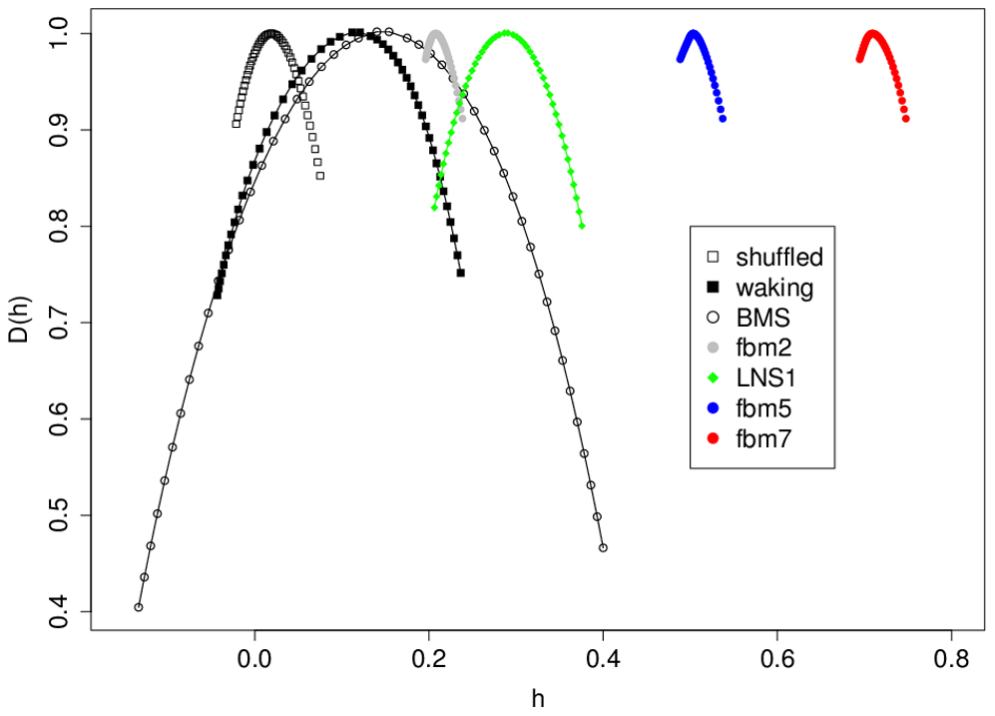
Comparison of MF-DFA spectrum from waking EEG to numerical models of mono- and multifractal processes. Data points represent individual D(h) and h values from MF-DFA from a single time series of each type. *waking*: waking EEG (8 m, n = 120,000) from a single subject; *shuffled*: waking EEG with values shuffled prior to MF-DFA analysis; *BMS*: binomial multifractal series model with a = 0.6 (n = 120,000; Kantelhardt et al, 2002 [33]); *LNS1*: log normal sigma 0.1 multifractal model data (n = 32,768; Arneodo et al 1998 [50]; http://www.physionet.org/physiotools/multifractal/); *fbm2, 5, 7*; fractional Brownian motion monofractal models with Hb values of 0.2, 0.5, 0.7 as indicated (n = 120,000 each; dvfBm 1.0 R package).

For each time series, both analyses produce spectra such as those shown in Fig. 1, each consisting of a set of 48 discrete points (h, D(h)) with inverted parabolic shape. For each spectrum we compute the parameters mean_h and mean_D(h), by averaging the points. We also calculate width_h as the difference between the maximum h and the minimum h and height_D(h) as the difference between the maximum D(h) and minimum D(h).

### Fractal Simulations

Fractional Brownian motion monofractal series were generated with Hurst exponent (H) values of 0.2, 0.5 and 0.7 using the dvfBm R package (120,000 data points each; version 1.0 [44]). The binomial multifractal series was used as described [33], where a series of N = 2^n_max_^ numbers with index k = 1,…, N, is defined by
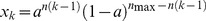
(2)


For this series, *a* is a user-defined parameter which can take values 0.5<*a*<1.We chose the parameter *a* = 0.6 such that the resulting multifractal spectrum roughly matches that of the MF-DFA spectra from the EEG samples. Here *n(k)* is the sum of digits equal to 1 in the binary representation of the index *k* (120,000 data points). As an example, choosing an index value of *k* = 13, *n*(13) = 3, as the binary representation of the decimal number 13 is 1101. The log normal sigma 0.1 multifractal series (32,768 data points) was downloaded from http://www.physionet.org/physiotools/multifractal/, made from the log-normal wavelet cascade algorithm with parameters *ν = ln(2)/4* and *σ = 0.1* as described [34].

### Statistics

**Figure 2:**
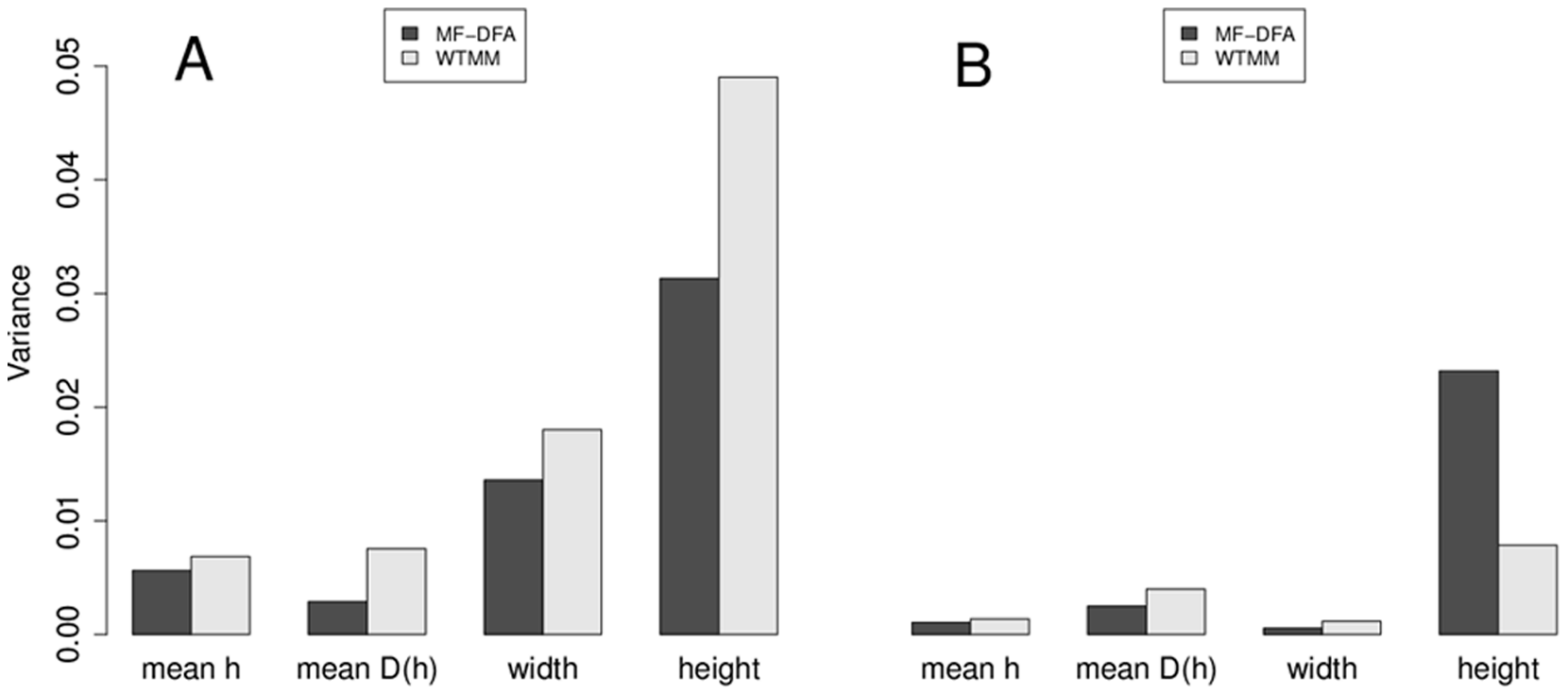
Variance comparison between MF-DFA and WTMM techniques. **A**. For 14 subjects with 8 m of waking EEG each, divided into 30 s segments (16 segments of n = 7500 data points each per subject), multifractal spectra were calculated (total of 224 segments). Mean Hölder exponent value (mean_h), width of the Hölder exponents (width_h), mean fractal dimension (mean_D(h)) value, and height of the multifractal singularity spectrum (height_D(h)) were calculated for each segment. **B**. The 14 subjects’ 8 m of waking EEG were analyzed whole, and multifractal specta were calculated. Note the trend to reduced variance with increasing length of EEG tracing.

All statistical tests were performed using R (v 2.13; [R Core 42]) and IBM SPSS (v.21.0, IBM, 2012). General linear modeling was conducted using IBM SPSS, adjusting for subject-level effects. For the results presented for the Figure 2 data, we estimated the expected standard deviation of the sample variances for each of the two techniques as described [45], using the formula:
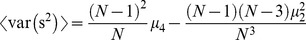
(3)


Where *N* is the sample size, and *µ_2_* and *µ_4_* are the second and fourth central moments of the distribution, respectively. We estimate *µ_2_*, the true variance of the distribution as the sample variance, and calculate *µ_4_* as:
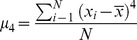
(4)


We compared the root mean square error of the combined expected standard deviations for both MF-DFA and WTMM techniques to the measured difference in variance to assess how meaningful the measured differences were likely to be. As a rule of thumb, if the measured difference in the variances was greater than twice the pooled expected standard deviation of the variances, this would imply that there was likely to be a true difference between the measured variances for each technique.

## Results

### Modeling Human EEG as a Multifractal Process using MF-DFA

In order to assess the feasibility of using MF-DFA analysis on human EEG tracings, we performed MF-DFA on time series derived from 8 m long EEG tracings from subjects in the MIT-BIH slpdb database annotated for the waking state of consciousness (typical example from one subject presented in Figure 1). For each time series this analysis produced an MF-DFA spectrum of typical inverted parabolic shape with width_h invariably ≥0.21 units (Figure 1; Table 2). Shuffling of the EEG time series followed by MF-DFA abolishes the multifractality (Figure 1), resulting in a monofractal spectrum with mean_h of 0. In order to compare spectra derived from EEG with spectra derived from well-understood monofractal (fractional Brownian motion (fBm)) and multifractal series, we also performed the MF-DFA analysis on various fractal simulations (Figure 1). In all cases, the MF-DFA of fBm generated a narrow MF-DFA spectrum (<0.1 units), consistent with monofractality. By contrast, MF-DFA of both the binomial multifractal series and the log normal sigma multifractal series generated wider spectra (larger width_h) with a larger range of D(h) (larger height_D(h)) than the monofractal series (figure 1). By direct comparison, MF-DFA spectra of human waking EEG appear to have a degree of multifractality in between the two multifractal simulations, and clearly greater than those for the monofractal simulations (Figure 1).

**Table 2:** Individual subject data for 8 min waking EEG MF-DFA spectra.

Subject	mean_h	width_h	mean_D(h)	height_D(h)	
S1	0.099	0.28	0.884	0.273	
S2	0.105	0.232	0.902	0.403	
S3	0.203	0.332	0.848	0.642	
S4	0.078	0.254	0.888	0.471	
S14	0.147	0.278	0.891	0.272	
S16	0.128	0.259	0.900	0.231	
S32	0.112	0.379	0.832	0.539	
S37	0.101	0.271	0.885	0.522	
S41	0.093	0.245	0.890	0.418	
S48	0.106	0.324	0.856	0.497	
S59	0.129	0.334	0.855	0.556	
S60	0.099	0.249	0.892	0.335	
S61	0.14	0.211	0.904	0.735	
S66	0.136	0.239	0.890	0.498	
**mean (s.d.)**	**0.12 (0.03)**	**0.278 (0.05)**	**0.880 (0.02)**	**0.456 (0.140)**	

In Table 2, we show the parameters derived from all 14 subjects’ MF-DFA analyses on 8 m long waking EEG tracings.

### Comparison of MF-DFA to WTMM Multifractal Spectra for EEG

To directly compare the variability of multifractal spectral results from MF-DFA to that for WTMM, we utilized a MIT-BIH slpdb dataset comprised of 16 segments of 30 s each (7500 datapoints) of waking EEG derived from 14 subjects, and performed both types of multifractal analyses on each segment (Figure 2A). For each multifractal spectrum from each segment, we calculated mean_h, mean_D(h), width_h, and height_D(h) (Figure 2A). WTMM and MF-DFA spectra were comparable overall (cf. [36]; WTMM spectrum data not shown). The variances for MF-DFA were markedly decreased compared to those for WTMM. We calculated an estimate of the pooled estimated standard deviation for the calculated sample variances for each measure, and compared this to the difference in sample variance between techniques to the pooled estimated standard deviation as a ratio. Using a cutoff of >2 standard deviations as a rough threshold for whether the measured difference in sample variances was likely to be meaningful, we found values of 1.3 for mean_h, 2.9 for width_h, 7.6 for mean_D(h), and 4.2 for height_D(h). This indicates that the variances for the latter three measures were likely to be less for the MF-DFA technique than for the WTMM technique with 30 s EEG segments (Figure 2A).

**Figure 3:**
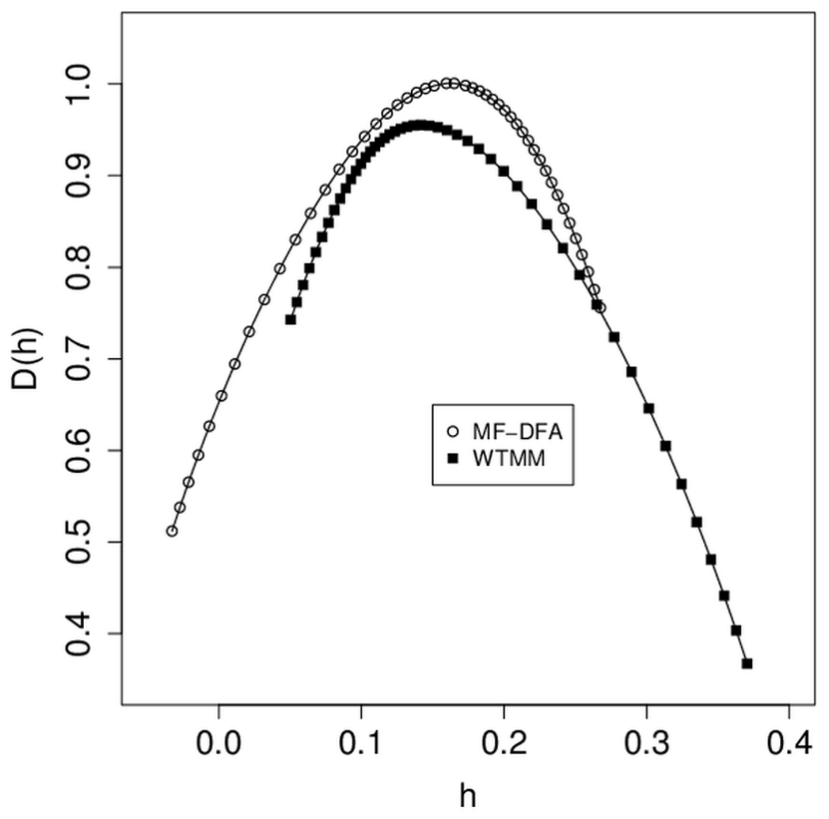
Comparison of waking EEG between MF-DFA and WTMM. For 14 subjects with 8 m of EEG from waking data per subject, MF-DFA and WTMM spectra were calculated for each 8 m EEG series. Average multifractal spectra for each technique shown here were calculated by averaging individual spectra across subjects: mean_h±s.d. is 0.12±0.03 for MF-DFA, and 0.17±0.04 for WTMM.

Next we repeated this analysis with the entire 8 m EEG from each of 14 subjects, by comparing the variances derived from mean_h, width_h, mean_D(h), and height_D(h) for the MF-DFA and WTMM techniques (Figure 2B). As expected, there was a strong trend for decreased variance overall for the longer tracings (Figure 2B). Via the same estimation of the estimated standard deviation of the pooled estimated variances, compared to the measured difference in sample variances, we found values of 0.6 for mean_h, 0.4 for width_h, and 2.0 for mean_D(h), indicating that of these three measures, only the variance in mean_D(h) was likely to be lower for MF-DFA than for WTMM. By contrast, for the height_D(h) measure, we found a ratio of 3.3, indicating that for the 8 m tracing, the WTMM variance was likely to be lower than that for MF-DFA (Figure 2B). We have plotted mean multifractal spectra from the entire 8 m EEG from each of 14 subjects for the MF-DFA and WTMM techniques to provide for a graphical comparison of the results given from each analysis (Figure 3).

### Linear Model Comparison of MF-DFA Spectra between Waking and Sleep Stage 2

**Figure 4:**
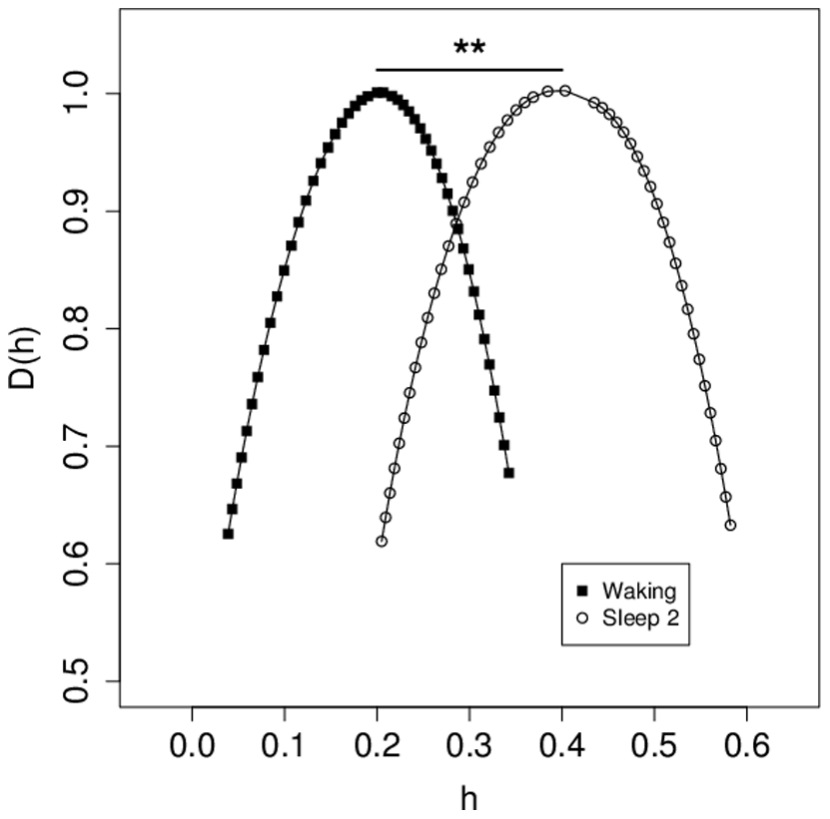
Comparison between MF-DFA spectra of from waking and sleep stage 2. For 14 subjects with 8 m of EEG from both waking and sleep stage 2 per subject, EEG was divided into 16 segments of 30 s each, and MF-DFA spectra were calculated for each segment (224 segments for each state of consciousness). Average MF-DFA spectra for each consciousness state shown here were calculated by averaging across individual spectrum values for each subject. **: p<0.001 for effect of state of consciousness by general linear modeling based on mean_h.

Given that the MIT-BIH slpdb dataset had the best representation among subjects for waking and sleep stage-2 EEG data [41], we utilized a dataset of EEGs derived from 14 subjects comprised of 16 segments of 30 s of EEG (7500 datapoints) per subject which had been annotated for both waking and sleep stage-2 EEG. Average MF-DFA spectra for all segments are plotted in Figure 4. We also computed MF-DFA spectra for each segment, and linear modeling was used to perform comparisons between these states of consciousness, using data for mean_h, mean_D(h), width_h, and height_D(h) separately (Figure 4). For mean_h, there was a large difference between states of consciousness, with waking having smaller mean_h values (F_(1,433)_ = 671, p<0.001). By contrast, there were no differences between sleep stages on width_h, mean_D(h) and height_D(h).

### Comparison of MF-DFA Spectra across All Sleep Stages

**Figure 5:**
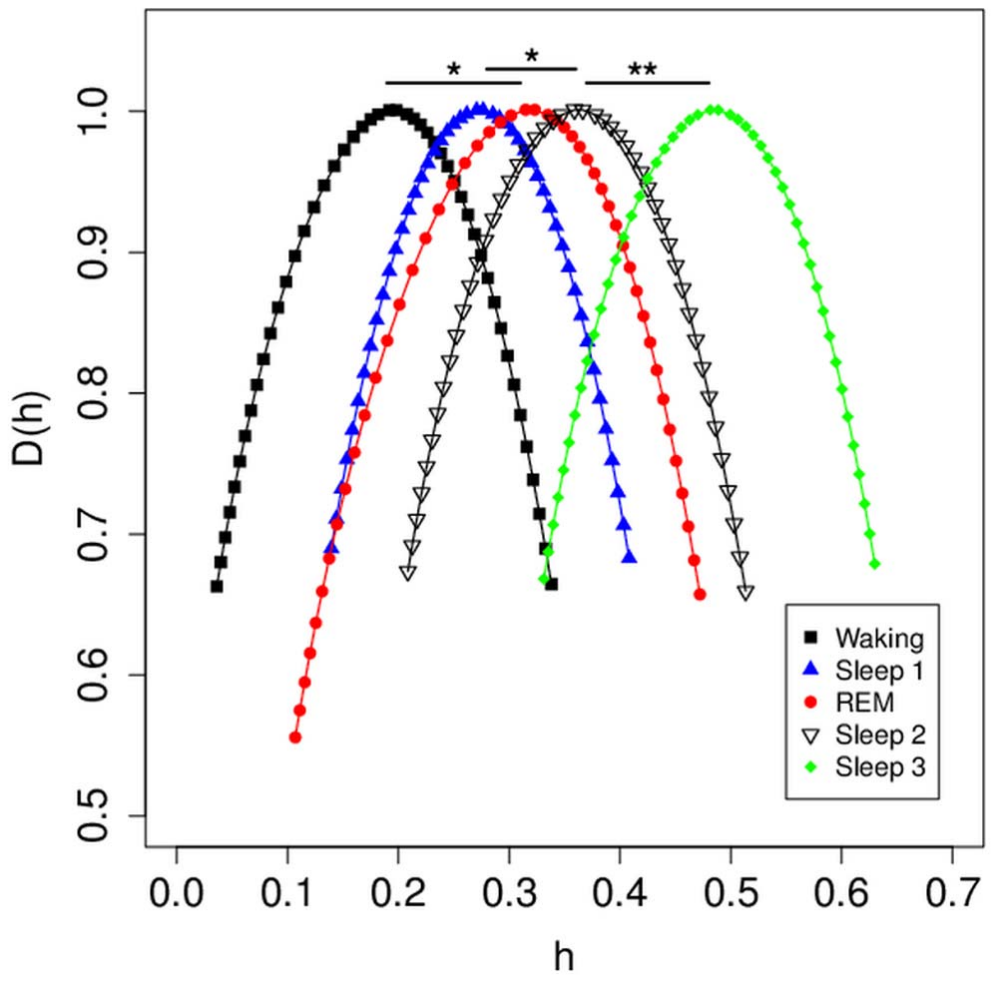
Comparison among stages of sleep for 1 minute of EEG data. For each stage from each subject, 1 min of EEG data was used to calculate MF-DFA spectra. Average MF-DFA spectra for each consciousness state shown here were calculated by averaging across individual spectrum values for each subject. Mean h values were then calculated for the h range, and differences between sleep stages compared by paired t testing. *p<0.05; **p<0.01. Significant differences were found for the waking-REM, Sleep 1-Sleep 2, and Sleep 2-Sleep 3 comparisons.

Given the sensitivity of MF-DFA to detect differences among different states of consciousness within subjects with a larger dataset (Figure 4), we decided to test the ability of MF-DFA mean_h values to distinguish among states of consciousness with only a single minute of EEG recording, across varying numbers of subjects (as not all subjects had good quality EEG data for each state of consciousness). We used 1 m (15000 datapoints) of annotated EEG data from subjects with waking (n = 14), REM (n = 12), sleep stage 1 (n = 9), sleep stage 2 (n = 15), and sleep stage 3 (n = 8). For each EEG trace, MF-DFA spectra were calculated, and MF-DFA spectra averaged across subjects for each state of consciousness are plotted in Figure 5. We paired t tests to assess for differences between mean_h values for each state of consciousness (Figure 5). There are significant differences for mean_h values between waking and REM EEGs (t(10 = 2.8, p = 0.0018), sleep stage 1 and sleep stage 2 EEGs (t(8) = 2.92, p = 0.019), and sleep stages 2 and sleep stage 3 EEGs (t(7) = 4.97, p = 0.005) (Figure 5).

## Conclusions

### Human EEG is Well-modeled as a Multifractal Process using MF-DFA

Given that finite-size effects result in a level of uncertainty in the calculation of MF-DFA spectra (and indeed, all techniques to estimate multifractality), care must be taken to ensure that MF-DFA results are consistent with true multifractality [24], [33], [46]. This can be done by comparing results from multifractal analysis with a given time series with that from simulations from mono- and multifractal series (see Figure 1). MF-DFA results from human EEG are comparable to those from known multifractal processes, and appear to have a larger degree of multifractality than known monofractal processes (Figure 1; also cf. [35]–[37]). Recent calculations of the degree of finite-size effects to be expected in multifractal spectrum calculations for non-multifractal processes also support these results, in that we see a larger degree of multifractality than expected by finite-size effects alone [46]. Taking into account the results presented here, results from previously published data [35]–[37], and results from numerical simulations [24], [33], [46], these data support the hypothesis that human EEG can be successfully modeled as a multifractal process, which may provide additional insight into changes in brain neuronal dynamics associated with pathological states. To our knowledge, these results are the first to formally assess the suitability of multifractal methods to human EEG using simulations of multifractal and monofractal data. Indeed, investigators using WTMM have already shown differences in multifractal spectra associated with psychiatric symptoms among subjects [37].

### MF-DFA may be More Consistent than WTMM with Shorter EEG Tracings

MF-DFA is an established technique for assessing multifractality, which has been used successfully in several different types of analysis, from simulations [33], to geophysics and ion channels [38], to hydrology [47], to cardiology [43]. It has been described as being comparable in terms of results, but needing less computational power than WTMM [24], [33]. Our results reported here suggest that MF-DFA may be more consistent than WTMM in terms of having a lower variance for parameters determined from multifractal spectral data for shorter recordings (30 s, or 7500 data points at 256 Hz, Figure 2A), but being roughly consistent with WTMM for longer (8 m) recordings (Figure 2B). These results are supported by previous reports showing that MF-DFA produces less overall variability than WTMM in other model systems as well, particularly with smaller data sets [33], [43], [48]. Therefore, MF-DFA may be superior to WTMM in detecting changes in neuronal dynamics underlying changes of consciousness or perception via EEG in shorter recordings of ∼30 s. However, it is interesting to note that while both techniques give similar results for a given EEG time series, they are certainly not identical (Figure 3). While both methods aim to estimate the multifractal spectrum, they use very different means to calculate singularities in data, and thus assess for multifractality in time series [32], [33]; therefore it is not surprising that the results of the two techniques may vary.

### MF-DFA may have Utility in the Recognition of Changes in States of Consciousness

Data presented here (Figures 3 and 4) support the notion that MF-DFA analysis of even relatively short (∼1 m) EEG tracings may have sufficient sensitivity to assist in automatic recognition of changes in the state of consciousness, including sleep stages in polysomnography. Comparing differences in mean_h values is likely to be the most useful technique, given that these tend to vary more between different states of consciousness than mean_D(h) and other values (Figures 4 and 5). In both the current study, and in the study of WTMM analysis of sleep stage differentiation [36], it is notable that only the mean_h values vary among sleep stages. It is interesting to note that both mean_h and width_h values were found to change within subjects after exposure to a painful stimulus [37], demonstrating that different states of consciousness may result in different effects on the multifractal spectrum. Current clinical criteria for sleep staging do not include computer-assisted feature detection, however the data presented here will add to emerging evidence that automatic sleep-stage detection could have a role to play in the future [49].

A significant limitation of this study is the limited publicly available dataset, with more than 20 year old EEG data, and minimal accompanying demographic information. Similarly, only a single EEG channel was provided, which differed among subjects. However, given the robust results we obtained in comparing multifractal spectra for different states of consciousness, it is clear that MF-DFA analysis of EEG tracings certainly deserves additional study with larger and more complete clinical EEG datasets.

Our results suggest that multifractal analysis via MF-DFA of EEG signals recorded from humans may be used to gain an improved understanding of the relevant underlying neuronal dynamics, compared to traditional techniques. Given that cortical neuronal networks exhibit nonlinear interactions characterized by a range of fractal exponents with varying scales, the technique of MF-DFA has the potential to be capable of describing essential features of the underlying neuronal dynamics for EEG signals in a way that is superior either to traditional techniques (e.g., spectral analysis via Fourier transform), or measures derived from monofractal analysis (e.g., monofractal box-counting methods or standard Detrended Fluctuation Analysis (DFA)). Brain disorders in humans are thought to reflect disorders of neuronal dynamics, and therefore multifractal DFA spectrum analysis of human EEG signals may prove to yield additional insights into disorders of neuronal dynamics than other currently available methods.
